# FcRγ-dependent immune activation initiates astrogliosis during the asymptomatic phase of Sandhoff disease model mice

**DOI:** 10.1038/srep40518

**Published:** 2017-01-13

**Authors:** Yasuhiro Ogawa, Takafumi Sano, Masahiro Irisa, Takashi Kodama, Takahiro Saito, Eiri Furusawa, Katsutoshi Kaizu, Yusuke Yanagi, Takahiro Tsukimura, Tadayasu Togawa, Shoji Yamanaka, Kohji Itoh, Hitoshi Sakuraba, Kazuhiko Oishi

**Affiliations:** 1 Department of Pharmacology, Meiji Pharmaceutical University, Tokyo, Japan; 2 Department of Functional Bioanalysis, Meiji Pharmaceutical University, Tokyo, Japan; 3 Department of Pathology, Yokohama City University School of Medicine, Yokohama, Japan; 4 Department of Medicinal Biotechnology, Institute for Medicinal Research, Graduate School of Pharmaceutical Sciences, The University of Tokushima, Tokushima, Japan; 5 Department of Clinical Genetics, Meiji Pharmaceutical University, Tokyo, Japan

## Abstract

Sandhoff disease (SD) is caused by the loss of β-hexosaminidase (Hex) enzymatic activity in lysosomes resulting from *Hexb* mutations. In SD patients, the Hex substrate GM2 ganglioside accumulates abnormally in neuronal cells, resulting in neuronal loss, microglial activation, and astrogliosis. *Hexb*^−/−^ mice, which manifest a phenotype similar to SD, serve as animal models for examining the pathophysiology of SD. *Hexb*^−/−^ mice reach ~8 weeks without obvious neurological defects; however, trembling begins at 12 weeks and is accompanied by startle reactions and increased limb tone. These symptoms gradually become severe by 16–18 weeks. Immune reactions caused by autoantibodies have been recently associated with the pathology of SD. The inhibition of immune activation may represent a novel therapeutic target for SD. Herein, SD mice (*Hexb*^−/−^) were crossed to mice lacking an activating immune receptor (*FcRγ*^−/−^) to elucidate the potential relationship between immune responses activated through SD autoantibodies and astrogliosis. Microglial activation and astrogliosis were observed in cortices of *Hexb*^*−*/*−*^ mice during the asymptomatic phase, and were inhibited in *Hexb*^−/−^ *FcRγ*^−/−^ mice. Moreover, early astrogliosis and impaired motor coordination in *Hexb*^*−*/*−*^ mice could be ameliorated by immunosuppressants, such as FTY720. Our findings demonstrate the importance of early treatment and the therapeutic effectiveness of immunosuppression in SD.

Sandhoff disease (SD, GM2 gangliosidosis 0 variant, OMIM #268800) is a glycosphingolipid storage disease caused by mutations in *Hexb* gene. Such mutations result in defective β-hexosaminidase (Hex) activity with GM2 gangliosides and related glycolipid accumulation in the lysosomes of neuronal cells. The accumulation of glycolipids leads to severe neurodegeneration via unknown pathological mechanisms. β-Hex consists of two major isoforms, β-Hex A (HexA; αβ heterodimer) and B (HexB; ββ homodimer), as well as one minor isoform, β-Hex S (HexS; αα homodimer). The human *Hexa* and *Hexb* genes encode the α- and β-subunits, respectively. *Hexb* mutations result in insufficient HexA and HexB enzyme activities, which ultimately result in SD. As HexA, but not HexB, can degrade GM2, the absence of HexA enzyme activity in brains of SD patients leads to progressive GM2 accumulation.

*Hexb*^−/−^ mice develop SD-like disease and serve as an animal model for dissecting the pathology of SD^1^,^2^,^3^. Previous studies have shown that *Hexb*^−/−^ mice exhibit no obvious neurological disorders until ~8 weeks of age; however, by 12 weeks of age, they develop tremors, startle reactions, and increased limb tone, and these symptoms become severe by 16–18 weeks of age^2^,^3^,^4^. Post-mortem dissections of SD patients have revealed gliosis in many regions, including the cerebral cortex and cerebellum, as well as significant nerve loss^5^. *Hexb*^−/−^ mice also exhibit various neurological symptoms at 16 weeks, including neuronal loss in the thalamus, brainstem, and spinal cord, which have been associated with apoptotic signals^6^,^7^. However, the mechanisms whereby the excessive accumulation of glycolipids eventually results in neurological disorders have not yet been elucidated in detail.

Microglia/macrophage-mediated inflammatory processes in the central nervous system (CNS) have been implicated in glycosphingolipid storage diseases, including GM1 and 2 gangliosidosis, and possibly in neurodegenerative processes^8^,^9^,^10^,^11^. Previous studies showed that the inflammatory responses in the CNS caused nerve damage and rapidly induced neuronal apoptosis through the expression of inflammatory mediators in SD^11^,^12^. Resting ramified microglia are readily activated and convert into amoeboid state under certain CNS pathologies. Although activated microglia play a role in neuron survival and neurogenesis by releasing neurotropic and anti-inflammatory factors^13^,^14^, they are also involved in the neuronal injury by releasing pro-inflammatory factors such as interleukin-1 (IL-1) and tumor necrosis factor alpha (TNF-α). Wada *et al*. reported that activated amoeboid microglia could be identified at 2 months of age and were increased in number at 3 months of age in parenchyma of the spinal cord, brainstem, and thalamus in *Hexb*^−/−^ mice^12^. At the same time as that of activated microglia, TNF-α levels were elevated by 2-, 7-, and 15-fold over control mice at 2, 3, and 4 months of age, respectively. No apoptotic neurons were detected at 1 and 2 months of age. Apoptotic neurons could be identified at 3 months of age and were increased in number at 4 months of age in parenchyma of the spinal cord, brainstem, and thalamus. In addition to microglia, astrocytes also respond to various CNS pathologies through a process referred to as reactive astrogliosis^15^,^16^. Inflammatory cytokines released by activated microglia play an important role for the conversion of astrocytes into a reactive state^17^,^18^,^19^. Reactive astrocytes participate in regulation of inflammatory responses and play a role in the progression and severity of neurological disorders through the release of neurotoxic and pro-inflammatory factors^15^,^20^,^21^,^22^. Therefore, both activated microglia and astrogliosis are key components of the CNS immune responses.

Fc receptors (FcRs) are expressed on immune effector cells such as lymphocytes, macrophages, and mast cells, and play an important role in regulating immune responses^23^. In the CNS, FcRs are expressed on microglia, astrocytes, oligodendrocytes, and neurons, and involved in mediating immune reactions in the brain^24^. Excessive activation of FcRs in the CNS may participate in neurodegenerative and immunological diseases. There are at least three types of FcγRs; FcγRI, and FcγRIII. Both FcγRI and FcγRIII are composed of multimeric subunits including the Fc receptor common γ chain (FcRγ) that contains an intracellular tyrosine-based activating motif (ITAM), whose activation triggers phagocytosis, cytotoxicity, and release of inflammatory cytokines. In contrast, FcγRII is a single subunit receptor, which contains an immune tyrosine-based inhibitory motif and abrogates ITAM-mediated inflammatory responses^25^. A balance between activated and inhibitory FcRs signaling regulates immune responses. FcRγ-deficient mice are deficient for all activating FcγRs, whereas the inhibitory receptor FcγRII expression is unaltered^26^. Recent studies using FcRγ-deficient mice (*FcRγ*^−/−^) indicated that the production of autoantibodies followed by FcRγ-dependent autoimmune response play an important role in the pathophysiology of SD^8^. Yamaguchi *et al*. reported improved clinical symptoms, increased life span, and a decreased number of apoptotic cells following the disruption of *Fcrγ* in *Hexb*^−/−^ mice. The level of ganglioside accumulation, however, did not change. These reports raise the possibility that ablation of autoimmune responses may represent a novel therapeutic target for SD.

Herein, we examined the relationship between immune responses and glial cell activation by autoantibodies in the brain of mice with SD-like pathologies. We investigated whether reactive gliosis was induced in the CNS by innate immune cell activation using double-knockout mice (*Hexb*^−/−^ *FcRγ*^−/−^). Our present study shows that astrogliosis in *Hexb*^−/−^ mice is caused by FcRγ-dependent immune responses during the asymptomatic phase. Moreover, early astrogliosis and impaired motor coordination in *Hexb*^*−*/*−*^ mice could be ameliorated by immunosuppressants. Our findings demonstrate the importance of early treatment and the therapeutic effectiveness of immunosuppression in SD.

## Results

### HexB-deficient mice reflect the pathology of Sandhoff disease

*Hexb*^−/−^ mice showed no obvious symptoms by 8 weeks of age, when they were considered to be in the asymptomatic phase. However, they gradually developed bradykinesia and ataxia (e.g., gait disturbance) by 12 weeks of age, and exhibited neurological symptoms, such as epilepsy-like seizures, and died by 16–18 weeks of age. Therefore, we investigated whether these characteristics could be observed in *Hexb*^−/−^ mice.

**Figure 1:**
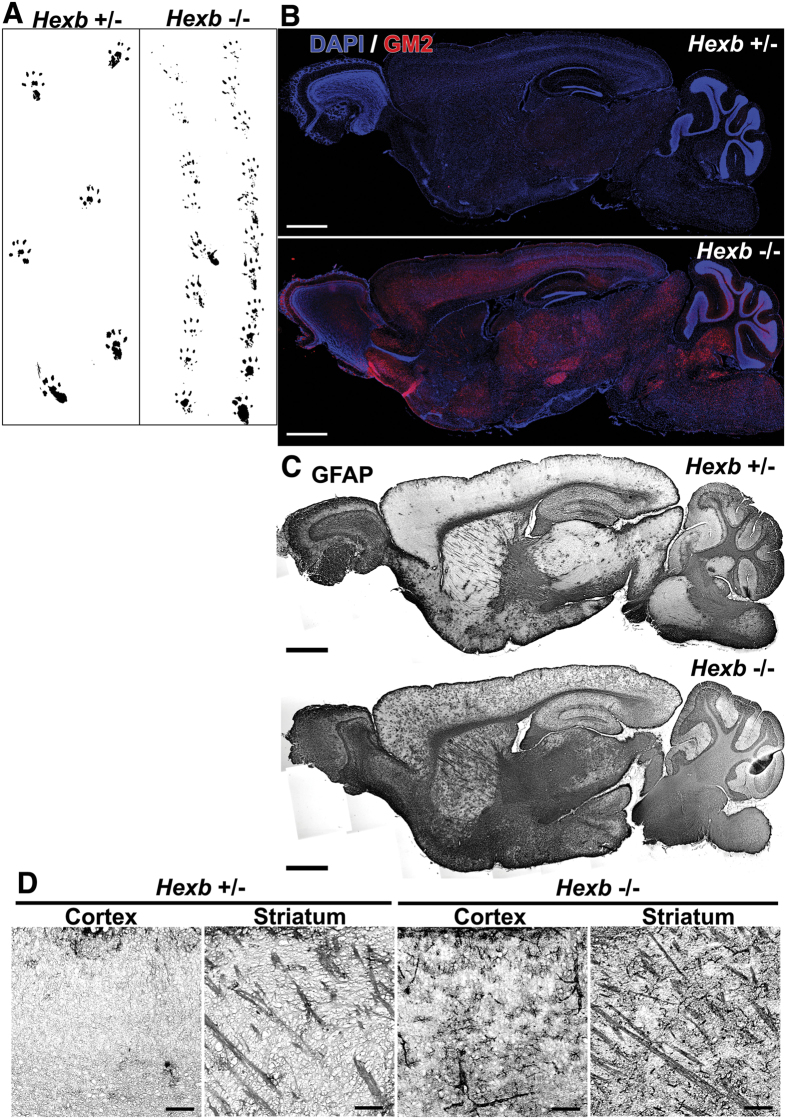
Phenotypes of *Hexb*^−/−^ mice at 16 weeks. (**A**) A *Hexb*^−/−^ mouse show a walking disability not observed in a heterozygote mouse. (**B**) The accumulation of GM2 (red) was detected in the whole brains of *Hexb*^−/−^ mice. Blue represents DAPI staining. (**C**,**D**) GFAP-immuno-signals were more strongly detected in the cortex and striatum of *Hexb*^−/−^ mice than heterozygote mice. Scale bar, (**B**,**C**) 1 mm; (**D**) 100 μm.

In the gait analysis, we assessed potential disorders of the hind legs in 16-week-old *Hexb*^−/−^ mice. The footprint width of *Hexb*^+/−^ mice was similar to their body width, whereas that of *Hexb*^−/−^ mice was smaller, and slight limping was noted (Fig. 1A). Brain sagittal sections from *Hexb*^−/−^ mice have been histologically examined. We observed extensive accumulation of GM2 in *Hexb*^−/−^ mice, but not in *Hexb*^+/−^ mice, which served as a control (Fig. 1B). Glial fibrillary acidic protein (GFAP) is an intermediate filament protein specifically expressed in astrocytes in the CNS. Increased level of GFAP represents astroglial activation and gliosis during neurodegeneration^27^. Only a few GFAP-positive activated astrocytes could be detected in the brains of *Hexb*^+/−^ mice, whereas they were more abundant and even located in the cerebral cortices and striata of *Hexb*^−/−^ mice, where only a very low GFAP signal is normally detected (Fig. 1C,D). Overall, the accumulation of GM2 and activated astrocytes could detect throughout almost the entire brains of *Hexb*^−/−^ mice.

Microarray analysis was subsequently conducted on brain tissues from 16-week-old *Hexb*^−/−^ mice using tissues from *Hexb*^+/−^ mice as a control. The mRNA expression levels of chemokine genes (e.g., *Ccl3*) and inflammatory cytokine- and complement-related genes (e.g., *Ifit3, Il1α, C3ar1*, and *C4b*) were increased in the brains of 16-week-old *Hexb*^−/−^ mice (Supplementary Table S1). Furthermore, genes known to be expressed by macrophages, such as Fc receptors (*Fcgr2b, Fcgr3*, and *Fcer1g*), *Cd68, Gpr84* (G protein-coupled receptor 84), and *Mpeg1* (macrophage expressed gene 1), were also up-regulated. The expression levels of *GFAP* and genes related to neuroinflammation were also elevated. Additionally, reduced levels of expression of *Cd209f* and *Cd209a* (associated with anti-inflammatory macrophages), myelin-associated proteins, *Mal* (myelin and lymphocyte protein, T-cell differentiation protein), and *Opalin* (oligodendrocytic myelin paranodal and inner loop protein) were detected. Furthermore, the mRNA expression levels of *Gpr37*, which is known to be an autism-related gene, were reduced (Supplementary Table S2)^28^.

### SD-related immune reactions involve FcRγ in *Hexb* ^−/−^ mice

Immunostaining revealed astrocyte activation in whole brains of *Hexb*^−/−^ mice. In support of these observations, our microarray analysis showed increased expression of genes, such as *Cd68* and *GFAP*, associated with the activation of microglia and astrocytes. Therefore, brain tissues from 16-week-old *Hexb*^+/−^, *Hexb*^*−*/*−*^, and *Hexb*^*−*/*−*^ *FcRγ*^−/−^ mice were subjected to immunostaining to determine whether activation of microglia and astrocytes was inhibited by the genetic ablation of FcR. Additionally, expression levels of inflammatory cytokines known to be induced by immune reactions through autoantibodies were analyzed by RT-PCR.

Microglial and astroglial activation was examined in the cerebral cortices of 16-week-old *Hexb*^−/−^ mice. CD68-positive microglia could be observed in 16-week-old *Hexb*^−/−^ mice, whereas only a few CD68-positive activated microglia could be detected in *Hexb*^+/−^ mice. In *Hexb*^+/−^ mice, Iba1-positive microglia were detected, and this number was significantly increased in *Hexb*^−/−^ mice. Iba1-positive microglia were reduced in double-knockout mice compared with *Hexb*^−/−^ mice, but not statistically significant (Supplementary Fig. S1). Astroglial activation was also examined in motor cortices of 16-week-old *Hexb*^−/−^ mice (Supplementary Fig. S2). In the cerebral cortices of 16-week-old *Hexb*^+/−^ mice, GFAP-positive astrocytes could be observed, and this number was significantly increased in the *Hexb*^*−*/*−*^ mice. In double-knockout mice, levels of GFAP-positive astrocytes were significantly lower than those observed in *Hexb*^*−*/*−*^ mice. Thus, brains of 16-week-old *Hexb*^*−*/*−*^ mice showed immune reactions mediated through FcRγ and consequent immune response-induced astrogliosis.

The expression levels of inflammatory cytokines were examined in brains of 16-week-old *Hexb*^−/−^ and double-knockout mice by quantitative RT-PCR. Levels of *IL-1α, IL-1β*, and *TNF-α* mRNA transcripts were significantly higher in *Hexb*^−/−^ than in *Hexb*^+/−^ mice, with levels of *IL-1β* and *TNF-α* being four- and five-fold greater, respectively. To examine the effects of suppressing immune responses through genetic ablation *Fcrγ* on the levels of these cytokines, double-knockout mice were compared with *Hexb*^−/−^ mice. We found that the expression levels of *IL-1α* and *IL-1β* were significantly reduced in double-knockout mice (Supplementary Fig. S3). The mRNA transcript levels of *IL-6* were not changed, while those of *TNF-α* showed a trend to be decreased that did not reach our threshold for statistical significance. Thus, production of some inflammatory cytokines could be suppressed by crippling the immune response through *Fcrγ* ablation.

### Immune responses during the asymptomatic phase involve FcRγ

Gliosis derived from activation and proliferation of microglia and astrocytes of 16-week-old *Hexb*^−/−^ mice could be reduced by suppressing autoantibody-mediated immune responses by deleting *Fcrγ*. Thus, to determine the time of gliosis, activation states of microglia and astrocytes were analyzed at 2–4 weeks of age by immunostaining. A similar analysis was also conducted in 4-week-old double-knockout mice to establish whether gliosis was caused by FcRγ-dependent immune responses.

**Figure 2:**
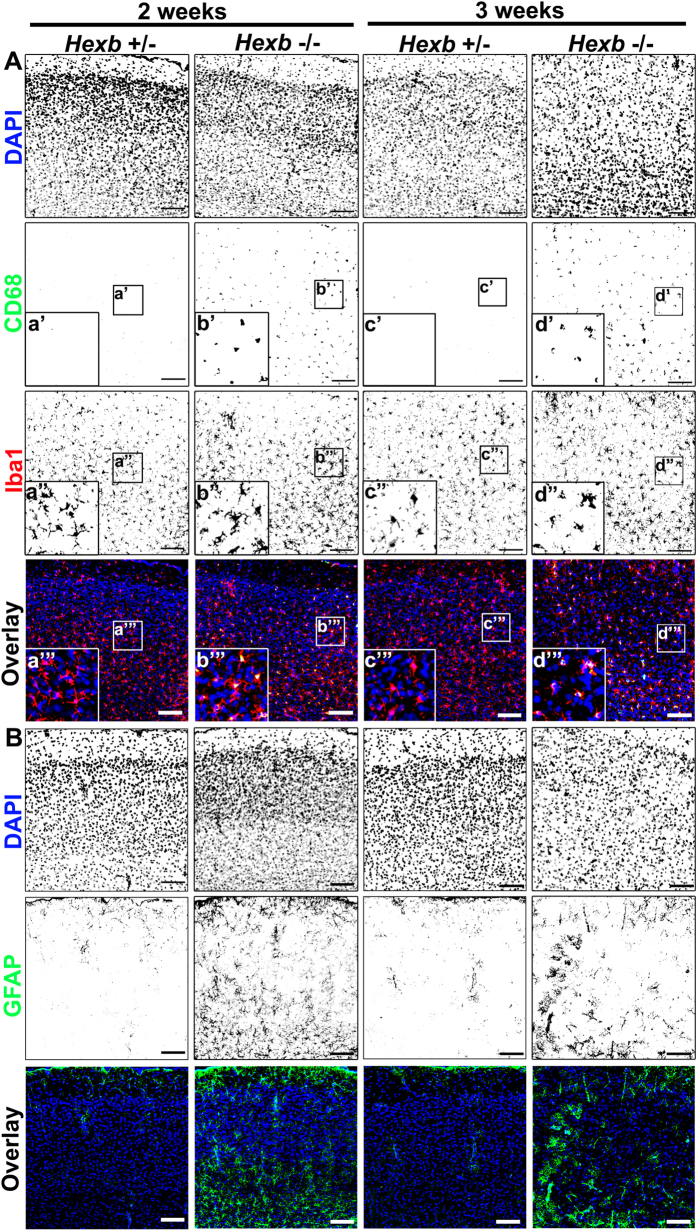
Microglial activation and astrogliosis in cortices of *Hexb*^−/−^ mice during development. (**A**) Immunostaining of coronal sections for CD68 (green) and Iba1 (red) in the cerebral cortices of *Hexb*^+/−^ and *Hexb*^−/−^ mice during development from 2 weeks to 3 weeks. (**B**) Immunostaining for GFAP (green) in the cerebral cortices of *Hexb*^+/−^ and *Hexb*^−/−^ mice during development from 2 weeks to 3 weeks. Insets (a–d) show magnified views of the boxed regions. Blue represents DAPI staining. Scale bar, 100 μm.

**Figure 3:**
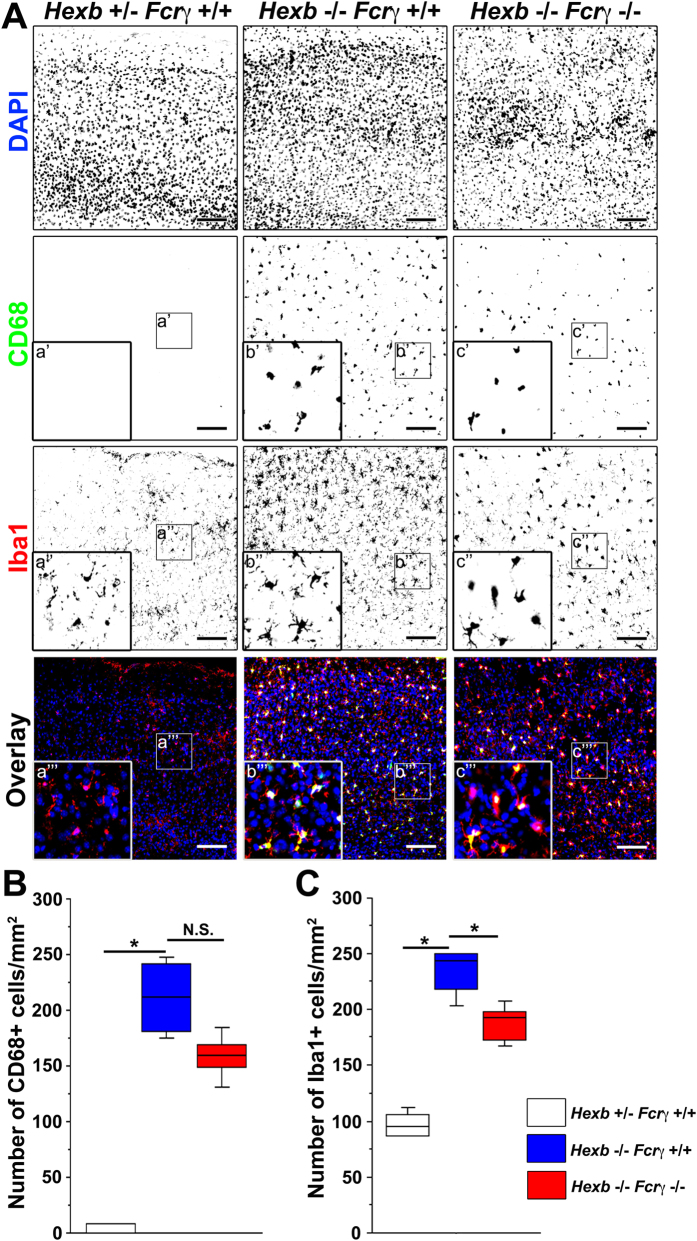
Reduction in microglial activity in the cortices of *Hexb*^−/−^ *FcRγ*^−/−^ mice at 4 weeks. (**A**) Immunostaining of coronal sections for CD68 (green) and Iba1 (red) in the cerebral cortices of *Hexb*^+/−^ *FcRγ*^+/+^, *Hexb*^−/−^ *FcRγ*^+/+^, and *Hexb*^−/−^ *FcRγ*^−/−^ mice at 4 weeks. Blue represents DAPI staining. Insets (a–c) show magnified views of the boxed regions. Scale bar, 100 μm. (**B**,**C**) Quantitative analysis for the number of CD68+ (**B**) and Iba1+ (**C**) cell immune signals in the cerebral cortices of *Hexb*^+/−^ *FcRγ*^+/+^, *Hexb*^−/−^ *FcRγ*^+/+^, and *Hexb*^−/−^ *FcRγ*^−/−^ mice at 4 weeks. Boxes, 25^th^–75^th^ percentile with the median indicated; bars, 10^th^ and 90^th^ percentiles. Analyzed using a Kruskal–Wallis test (nonparametric ANOVA) followed by a Dunn’s post hoc test (n = 5). *P < 0.05. N.S.: difference not significant.

Microglial activation was analyzed in the motor cortices of 2–3-week-old *Hexb*^−/−^ mice by immunostaining. Iba1-positive microglia could be observed in 2–3-week-old *Hexb*^+/−^ mice, whereas only a few CD68-positive activated microglia could be detected. By contrast, microglia with robust CD68-positive signals were activated in 2-week-old *Hexb*^−/−^ mice, and these cells were also observed in 3-week-old mice (Fig. 2A, Supplementary Fig. S4A). Subsequently, we investigated whether microglia were activated at the same time as astrocytes. We found that many GFAP-positive astrocytes could be detected in the motor cortices of *Hexb*^−/−^ and *Hexb*^+/−^ mice at 2 weeks of age. GFAP-positive astrocytes were detected in layers I and VI, but were less prominent in layers II–V and in the motor cortices of 3-week-old *Hexb*^+/−^ mice. Strikingly, few GFAP-positive astrocytes were observed in layers I–VI of the motor cortices of *Hexb*^−/−^ mice (Fig. 2B, Supplementary Fig. S4B). Thus, the number of CD68/Iba1-positive microglia were counted in the cerebral cortices of 4-week-old *Hexb*^+/−^, *Hexb*^−/−^, and double-knockout mice to determine whether immune responses that signal through FcRγ were involved in microglial activation (Fig. 3). CD68-positive cells were not detected in 1-mm^2^ sections from *Hexb*^+/−^ mice, while 212 (179–243) [median (25^th^–75^th^ percentile)] cells per section were counted in the cerebral cortices of *Hexb*^−/−^ mice, which was higher than that observed in *Hexb*^+/−^ mice, but not statistically significant (Fig. 3B). In double-knockout mice, a median value of 159 (143–174) CD68-positive cells per section were detected, which was significantly lower than that observed in *Hexb*^−/−^ mice. For Iba1-positive cells, 95 (87–108) cells per 1-mm^2^ section were observed in *Hexb*^+/−^ mice, while significantly more [244 (214–250) per section] were detected in *Hexb*^−/−^ mice. In double-knockout mice, 193 (172–201) Iba1-positive cells per section were detected, which was significantly lower than that observed in *Hexb*^−/−^ mice (Fig. 3C). Thus, microglia were already activated in 4-week-old *Hexb*^−/−^ mice, and FcRγ promoted microglia activation.

**Figure 4:**
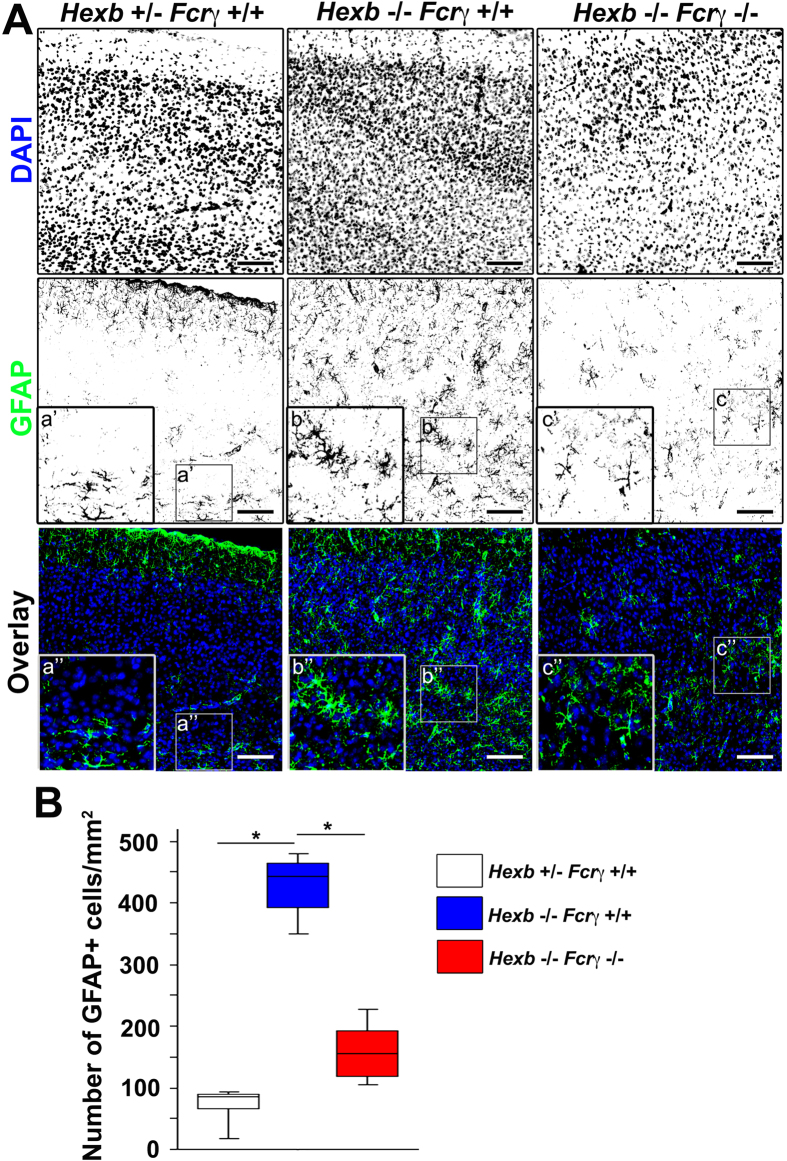
Reduction in reactive astrogliosis in the cortices of *Hexb*^−/−^ *FcRγ*^−/−^ mice at 4 weeks. (**A**) Immunostaining of coronal sections for GFAP (green) in the cerebral cortices of *Hexb*^+/−^ *FcRγ*^+/+^, *Hexb*^−/−^ *FcRγ*^+/+^, and *Hexb*^−/−^ *FcRγ*^−/−^ mice at 4 weeks. Blue represents DAPI staining. Insets (a–c) show magnified views of the boxed regions. Scale bar, 100 μm. (**B**) Quantitative analysis for the number of GFAP+ cell immune signals in the cerebral cortices of *Hexb*^+/−^ *FcRγ*^+/+^, *Hexb*^−/−^ *FcRγ*^+/+^, and *Hexb*^−/−^ *FcRγ*^−/−^ mice at 4 weeks. Boxes, 25^th^–75^th^ percentile with the median indicated; bars, 10^th^ and 90^th^ percentiles. Analyzed using a Kruskal–Wallis test (nonparametric ANOVA) followed by a Dunn’s post hoc test (n = 5). *P < 0.05.

Immunostaining to detect GFAP was conducted to examine the involvement of FcRγ-dependent microglial-mediated immune responses in astrocyte activation (Fig. 4), and we observed 443 (379–470) GFAP-positive astrocytes per 1-mm^2^ section in the cerebral cortices of 4-week-old *Hexb*^−/−^ mice, which was significantly greater than the 86 (50–90) astrocytes per section detected in *Hexb*^+/−^ mice. In the double-knockout mice, 156 (114–204) GFAP-positive astrocytes per section were observed, which was significantly lower than that observed in *Hexb*^−/−^ mice (Fig. 4B). In the brain, S100β is expressed abundantly in astrocytes and considered as an astrocyte marker. Double immunostaining for GFAP and S100β revealed that GFAP/S100β double-positive activated astrocytes detected in 4-week-old *Hexb*^+/−^ mice were markedly increased in *Hexb*^−/−^ mice (Supplementary Fig. S5). Thus, astrocytes were already activated in brains of 4-week-old *Hexb*^−/−^ mice, suggesting that this activation was partially attributed to microglial activation through FcRγ.

**Figure 5:**
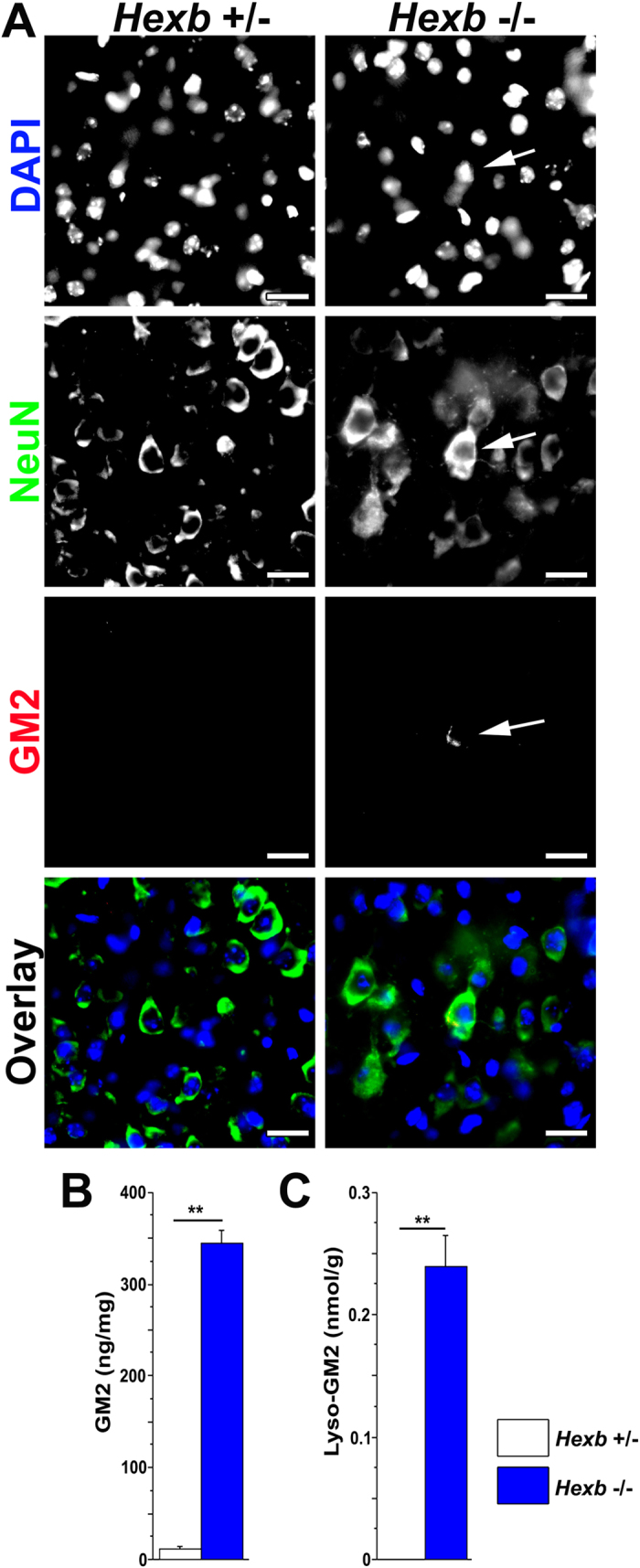
GM2 and Lyso-GM2 were up-regulated in cortices of *Hexb*^−/−^ mice at 4 weeks. (**A**) Immunostaining of coronal sections for NeuN (neuronal marker, green) and GM2 (red) in the cerebral cortices of *Hexb*^+/−^ and *Hexb*^−/−^ mice at 4 weeks. Arrows indicate NeuN/GM2 double-positive cells. Blue represents DAPI staining. Scale bar, 20 μm. (**B**,**C**), GM2 (**B**) and Lyso-GM2 (**C**) levels in the cerebral cortices of *Hexb*^+/−^ and *Hexb*^−/−^ mice at 4 weeks. Analyzed using the unpaired Student’s *t*-test. (*Hexb*^+/−^ mice; n = 4 and *Hexb*^−/−^ mice; n = 5). **P < 0.01.

Immunostaining for GM2 gangliosides, a hallmark of SD, revealed punctuate positive signals in cortices of 4-week-old *Hexb*^−/−^ mice (Fig. 5A). No positive signals were detected in *Hexb*^+/−^ mice. Measurements of GM2 and Lyso-GM2 levels in cerebral cortices of 4-week-old *Hexb*^+/−^ and *Hexb*^−/−^ mice by tandem mass spectrometry showed that the contents of GM2 and Lyso-GM2 in 4-week-old *Hexb*^−/−^ mice were significantly higher than those detected in *Hexb*^+/−^ mice (Fig. 5B,C).

### Early astrogliosis in *Hexb* ^−/−^ mice could be ameliorated by immunosuppressants

Astrogliosis developed in *Hexb*^−/−^ mice at 4 weeks of age, which we attributed to immune responses through FcRγ expressed by microglia. Therefore, we investigated whether astrogliosis could be improved by suppressing immune responses with immunosuppressive agents.

**Figure 6:**
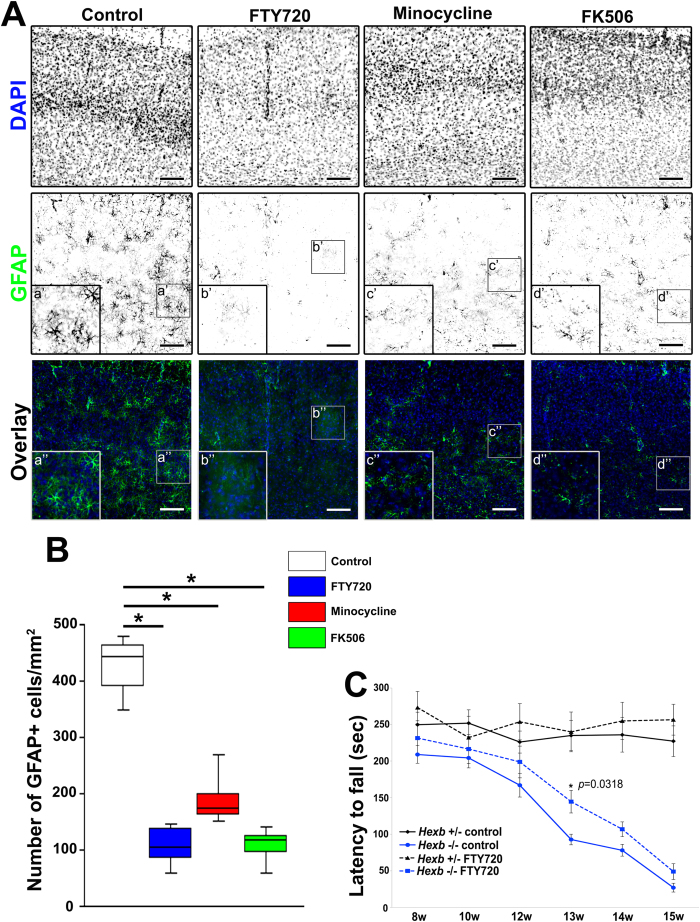
Reduction in reactive astrogliosis in cortices of immunosuppressant-treated *Hexb*^−/−^ mice at 4 weeks. (**A**) Immunostaining of coronal sections for GFAP (green) in the cerebral cortices of control, FTY720-, minocycline-, and FK506-treated *Hexb*^−/−^ mice at 4 weeks. Blue represents DAPI staining. Insets (a–d) show magnified views of the boxed regions. Scale bar, 100 μm. (**B**) Quantitative analysis for the number of GFAP+ cell immune signals in the cerebral cortices of control, FTY720-treated, minocycline-treated, and FK506-treated *Hexb*^−/−^ mice at 4 weeks. Boxes, 25^th^–75^th^ percentile with the median indicated; bars, 10^th^ and 90^th^ percentiles. Analyzed using a Kruskal–Wallis test (non-parametric ANOVA) followed by a Dunn’s post hoc test (n = 5). *P < 0.05. (**C**) Reduction in impaired motor coordination in FTY720-treated *Hexb*^−/−^ mice. FTY720 was orally administered to in *Hexb*^+/−^ or *Hexb*^−/−^ mice at 3–15 weeks (w) of age. The rotarod test revealed that the impaired motor coordination observed in *Hexb*^−/−^ mice was significantly reduced by the administration of FTY720 at 13 weeks. Analyzed using repeated-measures ANOVA followed by Tukey’s post hoc *t*-test. (n = 6). *P < 0.05; compared with untreated *Hexb*^−/−^ mice.

Diverse immunosuppressants (FTY720, minocycline, or FK506) with distinct mechanisms of action were intraperitoneally administered to in *Hexb*^+/−^ or *Hexb*^−/−^ mice at 3–4 weeks of age to examine effects on astrogliosis (Fig. 6A,B, Supplementary Fig. S5). A median value of 86 (50–90) GFAP-positive astrocytes per 1-mm^2^ section were observed in 4-week-old *Hexb*^+/−^ mice, while 443 (379–470) per section were detected in *Hexb*^−/−^ mice. After the administration of FTY720, minocycline, or FK506, this number was significantly reduced to 104 (79–141), 175 (161–223), and 118 (84–130), respectively (Fig. 6B). Thus, early astrogliosis in *Hexb*^*−*/*−*^ mice can be controlled by immunosuppressants.

### Impaired motor coordination in *Hexb* ^−/−^ mice could be ameliorated by FTY720

We investigated whether impaired motor coordination in *Hexb*^−/−^ mice could be improved by suppressing immune responses with FTY720. FTY720 was orally administered to in *Hexb*^+/−^ or *Hexb*^−/−^ mice at 3–15 weeks of age to examine effects on motor coordination activity (Fig. 6C). The rotarod test revealed that the time lapse was shortened at 12 weeks in *Hexb*^−/−^ mice compared with *Hexb*^+/−^ mice, and this deficiency was gradually increased for up to 15 weeks. Impaired motor coordination observed in *Hexb*^−/−^ mice was significantly reduced by the administration of FTY720 at 13 (*P* = 0.0318 vs. untreated *Hexb*^−/−^ mice) weeks. Thus, the deficit in motor coordination in *Hexb*^*−*/*−*^ mice can be improved by suppressing immune responses.

**Figure 7:**
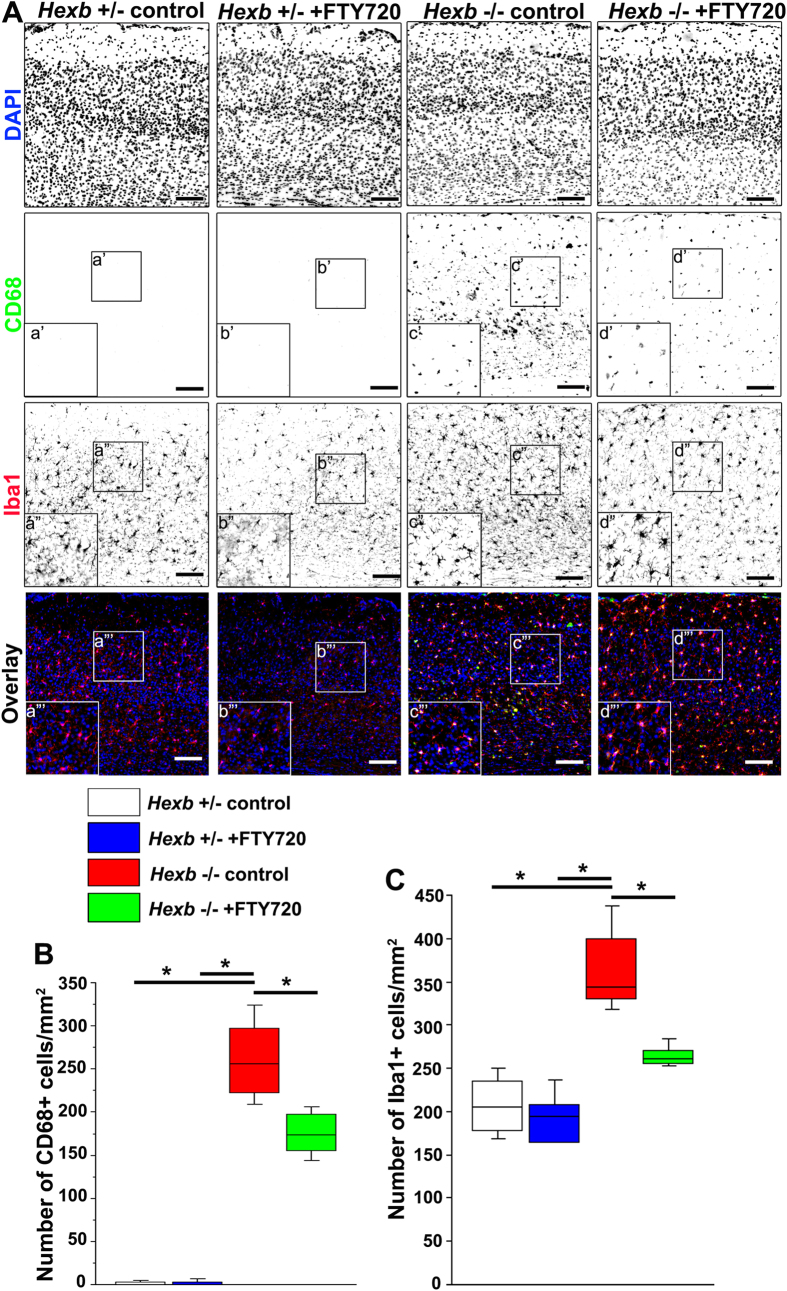
Reduction in microglial activity in cortices of FTY720-treated *Hexb*^−/−^ mice at 15 weeks. FTY720 was orally administered to *Hexb*^+/−^ or *Hexb*^−/−^ mice at 3–15 weeks of age. (**A**) Immunostaining of coronal sections for CD68 (green) and Iba1 (red) in the cerebral cortices of control and FTY720-treated *Hexb*^−/−^ mice at 15 weeks. Blue represents DAPI staining. Insets (a–d) show magnified views of the boxed regions. Scale bar, 100 μm. (**B**,**C**) Quantitative analysis of the number of CD68+ (**B**) and Iba1+ (**C**) cell immune signals in the cerebral cortices of control *Hexb*^+/−^, FTY720-treated *Hexb*^+/−^, control *Hexb*^−/−^, and FTY720-treated *Hexb*^−/−^ mice at 15 weeks. Boxes, 25^th^–75^th^ percentile with the median indicated; bars, 10^th^ and 90^th^ percentiles. Analyzed using a Kruskal–Wallis test (non-parametric ANOVA) followed by a Dunn’s post hoc test (n = 6). *P < 0.05.

Finally, brain tissues from 15-week-old mice were subjected to immunostaining to determine whether activation of microglia was inhibited by the administration of FTY720 at 3–15 weeks of age. CD68-positive cells were not detected in 1-mm^2^ sections from the cerebral cortices of *Hexb*^+/−^ mice (Fig. 7), while 256 (219–305) cells per section were counted in those of *Hexb*^−/−^ mice, which was significantly higher. In FYT720-treated *Hexb*^*−*/*−*^ mice, this number significantly decreased to 173 (152–200) per section (Fig. 7B). In *Hexb*^+/−^ mice, 205 (175–239) Iba1-positive microglia were detected per 1-mm^2^ section, while this number significantly increased to 344 (327–410) per section in *Hexb*^*−*/*−*^ mice. A total of 261 (254–273) Iba1-positive microglia could be observed per section in FYT720-treated *Hexb*^*−*/*−*^ mice, which was significantly reduced compared with untreated *Hexb*^*−*/*−*^ mice (Fig. 7C).

**Figure 8:**
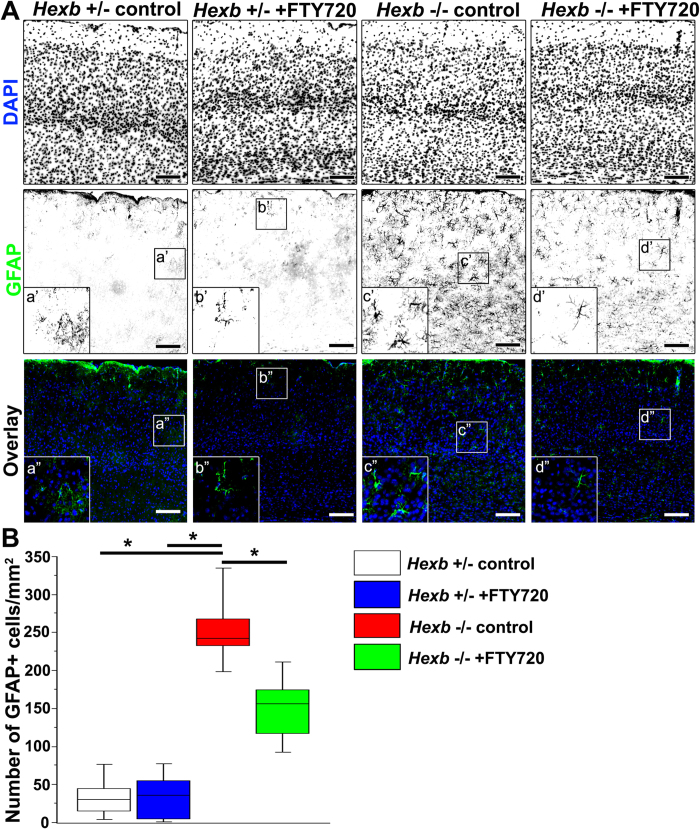
Reduction in reactive astrogliosis in cortices of FTY720-treated *Hexb*^−/−^ mice at 15 weeks. FTY720 was orally administered to *Hexb*^+/−^ or *Hexb*^−/−^ mice at 3–15 weeks of age. (**A**) Immunostaining of coronal sections for GFAP (green) in the cerebral cortices of control and FTY720-treated *Hexb*^−/−^ mice at 15 weeks. Blue represents DAPI staining. Insets (a–d) show magnified views of the boxed regions. Scale bar, 100 μm. (**B**) Quantitative analysis of the number of GFAP+ cell immune signals in the cerebral cortices of control *Hexb*^+/−^, FTY720-treated *Hexb*^+/−^, control *Hexb*^−/−^, and FTY720-treated *Hexb*^−/−^ mice at 15 weeks. Boxes, 25^th^–75^th^ percentile with the median indicated; bars, 10^th^ and 90^th^ percentiles. Analyzed using a Kruskal–Wallis test (non-parametric ANOVA) followed by a Dunn’s post hoc test (n = 6). *P < 0.05.

We subsequently investigated whether astrocyte activation could be inhibited by the administration of FTY720 (Fig. 8). In the cerebral cortices of *Hexb*^+/−^ mice, a median value of 30 (12–53) GFAP-positive astrocytes could be observed per 1-mm^2^ section, while they were significantly increased to 242 (223–286) per section in the *Hexb*^*−*/*−*^ mice. In FYT720-treated *Hexb*^*−*/*−*^ mice, the median value of GFAP-positive astrocytes was 156 (110–185) per section, which was significantly lower than that observed in untreated *Hexb*^*−*/*−*^ mice (Fig. 8B). These results show that the microglial activation and astrogliosis observed in the cortices of *Hexb*^−/−^ mice were inhibited by FTY720, suggesting a correlation between the severity of the neurological parameters and the severity of the histological abnormalities.

## Discussion

The relationship between immune responses and gliosis was examined using *Hexb*^+/−^ (negative control), *Hexb*^−/−^ (positive control), and *Hexb*^−/−^ *FcRγ*^−/−^ (double-knockout) mice. In addition to movement disorders, *Hexb*^−/−^ mice exhibited accumulation of GM2 and gliosis, the pathological hallmarks of SD, throughout brain tissues^8^. Thus, *Hexb*^−/−^ mice reproduce much of the pathology of SD and can serve as a model animal to examine the pathophysiology of SD.

The abnormalities observed in 16-week-old *Hexb*^−/−^ mice were examined by microarray analysis, which revealed increases in the expression levels of chemokine (e.g., *Ccl3*), inflammatory cytokine (e.g., *Ifit3* and *Il1α*), and complement-associated (e.g., *C3ar1*, and *C4b*) genes. Furthermore, the expression levels of macrophage-associated genes (*Fcgr2b, Fcgr3* and *Fcer1g, Cd68*, and *Gpr84*) were upregulated. The expression levels of *Gfap* and genes related to neuroinflammation were also markedly increased. Therefore, we examined the activation of glial cells in motor cortices by immunostaining. We then examined whether glial cell activation could be inhibited by the genetic ablation of *Fcrγ*. Yamaguchi *et al*. reported that the levels of accumulated GM2 in the brains of 14-week-old *Hexb*^−/−^ *FcRγ*^−/−^ mice did not change when compared with *Hexb*^−/−^ *FcRγ*^+/+^ mice^8^. Based on our microarray findings, CD68-positive microglial activation and GFAP-positive astroglial activation was studied. Activation of glial cells was reduced in double-knockout mice, suggesting that immune responses occurred via FcRγ and that astrogliosis was induced by immune responses that involved this receptor.

Expression levels of inflammatory cytokines in brain tissues of 16-week-old *Hexb*^−/−^ and double-knockout mice were subsequently examined by quantitative RT-PCR. The mRNA transcript levels of genes encoding IL-1α, IL-1β, IL-6, and TNF-α were significantly elevated in *Hexb*^−/−^ mice compared with *Hexb*^+/−^ mice. Thus, we investigated whether suppression of immune responses through the ablation of *Fcrγ* influenced these changes in cytokine expression levels by comparing double-knockout mice with *Hexb*^−/−^ mice. We found significantly reduced expression levels of *IL-1α* and *IL-1β* mRNA transcripts. IL-1β plays a role in memory impairment and locomotor activity, and has been implicated in the pathophysiology of neurological diseases^29^,^30^. No significant differences were noted in the expression levels of *IL-6*, whereas those of *TNF-α*, which encodes an inflammatory cytokine that is strongly involved in the pathology of *Hexb*^−/−^ mice, were slightly decreased. Abo-Ouf *et al*. reported that depletion of TNF-α in 17-week-old *Hexb*^−/−^ mice results in improved neurological function, decreased levels of astrogliosis, and reduced neural cell death^31^. Kyrkanides *et al*. previously reported that expression of *TNF-α* and microglial activation could be markedly reduced and apoptosis was suppressed by specifically inducing HexB enzyme expression in neurons of *Hexb*^−/−^ mice, whereas expression levels of *IL-1* and *Gfap* and the number of astrocytes remained unchanged^32^. Activated microglia are the main source of proinflammatory factors such as IL-1 and TNF-α^13^,^14^. In the present study, an increased number of CD68-positive activated microglia in 16-week-old *Hexb*^−/−^ mice were not significantly reduced in 16-week-old double knockout mice. Thus, TNF-α may be a strong driver of neuronal apoptosis that results from GM2 accumulation, but may play a less prominent role in FcRγ-dependent immune responses in 16-week-old *Hexb*^−/−^ mice.

Furthermore, the cerebral cortices of the aforementioned mice were analyzed at younger ages by immunostaining to identify microglial activation and the onset time of astrogliosis. We found that astrogliosis was present during the asymptomatic phase in 4-week-old *Hexb*^−/−^ mice, but was markedly reduced in double-knockout mice. Thus, FcRγ-dependent astrogliosis developed during the asymptomatic phase of SD. Chronic nerve inflammation begins developing during the asymptomatic phase and progresses to death in *Hexb*^−/−^ mice. Neither anti-GM2/-GA2 antibodies in the blood nor the accumulation of IgG in neurons were previously detected in *Hexb*^−/−^ mice at 4 weeks of age during the asymptomatic period^8^. However, Jeyakumar *et al*. reported that CD68-positive activated microglia disappeared from the brainstems of *Hexb*^−/−^ mice treated at 3 weeks of age with miglustat, an inhibitor of glucosylceramide synthase^9^, suggesting the presence of unmetabolized substrates in the brain during the asymptomatic phase that could activate microglia.

We also investigated whether astrogliosis could be suppressed by immunosuppressants such as FTY720 (fingolimod), minocycline, and FK506. FTY720 is an immunosuppressant, which modulates sphingosine 1-phosphate (S1P) receptors. FTY720 has a similar chemical structure to sphingosine, and can phosphorylated *in vivo* by sphingosine kinase to form FTY720-P [(S)-enantiomer], which can act as an agonist of 4 of the 5 S1P receptors (S1P_1_, S1P_3_, S1P_4_, and S1P_5_), excluding the S1P_2_ receptor. FTY720-P acts as an S1P receptor functional antagonist by causing their internalization. S1P receptors are expressed in astrocytes and microglia/macrophages^33^,^34^,^35^. Genetic deletion of either sphingosine kinase 1 or the S1P_3_ receptor in SD mice results in a reduced glial cell proliferation and astrogliosis^34^. FTY720 treatment has been shown to reduce microglial activation in cerebral ischemic lesions in mice^36^. FTY720 has also been shown to downregulate TNF-α and IL-1β produced by activated microglia and astrocytes^37^. We found that astrogliosis could be reduced by FTY720 treatment during the asymptomatic phase, suggesting that pathological conditions in *Hexb*^−/−^ mice may be improved through the inhibition of S1P receptor signaling. Minocycline, a tetracycline derivative with anti-inflammatory and immunosuppressive effects, mediates neuroprotection in experimental models of neurological diseases including cerebral ischemia, traumatic brain injury, and Huntington’s and Parkinson’s disease^38^,^39^,^40^,^41^,^42^. FK506 (tacrolimus), an immunosuppressant currently used in the clinic, is known to have neuroprotective and/or neuroregenerative activity in animal models of neurological diseases including traumatic brain injury, spinal cord injury, optic nerve crush, antiretroviral toxic neuropathy, Parkinson’s disease, and stroke^43^,^44^,^45^. Both minocycline and FK506 have been shown to inhibit microglial activation and production of inflammatory cytokines such as TNF-α and IL-1ß^46^,^47^. We found that astrogliosis could be reduced by either minocycline or FK506 treatment during the asymptomatic phase, suggesting that the inhibition of microglial activation and production of inflammatory cytokines may improve pathological conditions in *Hexb*^−/−^ mice.

Finally, we investigated whether impaired motor coordination in *Hexb*^−/−^ mice could be improved by suppressing immune responses with FTY720. We found that the deficiency could be reduced by FTY720, suggesting that the deficit in motor coordination in *Hexb*^*−*/*−*^ mice can be improved by suppressing immune responses through the inhibition of S1P receptor signaling. Brain tissues from 15-week-old mice were subjected to immunostaining to determine whether activation of microglia and astrocytes was inhibited by the administration of FTY720 at 3–15 weeks of age. The results showed that microglial activation and astrogliosis observed in the cortices of *Hexb*^−/−^ mice were inhibited by FTY720. This finding strongly supports the correlation between the severity of the neurological parameters and the severity of the histological abnormalities. Our findings demonstrate the importance of early treatment and the therapeutic effectiveness of immunosuppression in SD.

In summary, our present study shows that astrogliosis in *Hexb*^−/−^ mice is caused by FcRγ-dependent immune responses during the asymptomatic phase. Early astrogliosis and impaired motor coordination in *Hexb*^*−*/*−*^ mice could be ameliorated with immunosuppressants, such as FTY720. Our findings suggest the importance of early treatments with agents aimed at inducing immunosuppression. Such treatments might suppress the production of autoantibodies, resulting in a significant delay of the development of neurological symptoms. In the future, the effects of administering early-, mid-, and late-stage treatments with immunosuppressants on survival and motor functions should be analyzed in detail.

## Methods

### Mouse Models

All animal procedures were performed in accordance with the Guidelines for Animal Experimentation of the Japanese Association for Laboratory Animal Science, and were approved by the Institutional Animal Use and Care Committee of Meiji Pharmaceutical University. *Hexb*^−/−^ mice (C57BL/6 × 129sv) were kindly provided by Dr. Richard L. Proia (Genetics of Development and Disease Branch, National Institute of Diabetes, and Digestive and Kidney Disease, National Institutes of Health, Bethesda, MD, USA). *FcRγ*^−/−^ mice (C57BL/6 × 129sv)^26^ were bred with *Hexb*^−/−^ mice to obtain double-knockout mice (*Hexb*^*−*/*−*^ *FcRγ*^−/−^). Genotyping of these mice was determined by PCR as described by Yamaguchi *et al*.^8^. Male mice were used for all experiments except immunofluorescent staining of 2–3-week-old mice.

### Gait Analysis

The gaits of freely moving mice were tested on a confined walkway that was 30 cm wide ×40 cm long. After dipping hindpaws into black ink, mice walked on white paper to track each step. Upon completion of the test, the paper was scanned at 300 dpi.

### Antibodies

A mouse monoclonal antibody against GM2 ganglioside (GMB28; immunoglobulin M, 1:20) was kindly donated by Dr. Tai (Department of Tumor Immunity, The Tokyo Metropolitan Institute of Medical Science, Tokyo, Japan). An anti-GFAP (Dako, Carpinteria, CA, USA, 1:1000), anti-Iba1 (Wako Pure Chemical Industries, Osaka, Japan, 1:500), anti-CD68 (clone FA-11, AbD Serotec Ltd., Oxford, UK, 1:100), anti-NeuN (EMD Millipore, Billerica, MA, 1;1000) and anti-S100ß (GeneTex, Irvine, CA, 1:100) were used as primary antibodies. As secondary antibodies, Alexa-488-conjugated goat anti-mouse IgG (1:1000), Alexa-568-conjugated goat anti-mouse IgG (1:1000), Alexa-568-conjugated goat anti-mouse IgM (1:1000), Alexa-488-conjugated goat anti-rat IgG (1:1000), Alexa-488-conjugated goat anti-rabbit IgG (1:1000), Alexa-568-conjugated goat anti-rabbit IgG (1:1000) (all purchased from Molecular Probes, Eugene, OR, USA), and Histofine Simple Stain MAX-PO(R) (Nichirei Co., Tokyo, Japan) were used.

### Immunohistochemistry

Mice were deeply anesthetized and perfused with 4% paraformaldehyde in phosphate-buffered saline (PBS), pH 7.4. The brain was removed and post-fixed for a few hours in the same solution before being transferred into 20% sucrose in PBS. Brain tissues were then embedded in Tissue-Tek O.C.T. compound (Sakura Finetechnical, Tokyo, Japan) and frozen at −80 °C. Blocks were cut using a cryostat (Leica) to obtain 25-μm-thick sagittal or coronal sections, which were then placed onto glass slides (Platinum Pro Coat; Matsunami, Osaka, Japan). Sections were washed in PBS, treated with Histo-VT-one antigen retrieval reagent (Nacalai Tesque, Kyoto, Japan) for 20 min at 70 °C, washed in PBS, quenched in 0.3% H_2_O_2_ in MeOH for 30 min, and then incubated in blocking buffer (0.1% Triton X-100 and 10% normal goat serum in PBS) for 2 h. After blocking, sections were stained at 4 °C overnight with an anti-GFAP antibody. To detect primary antibody reactivity, sections were incubated for 2 h at room temperature with a peroxidase-conjugated goat anti-rabbit IgG antibody (Histofine Simple Stain MAX-PO(R)), and then were visualized using a Peroxidase Stain DAB Kit and an enhancer for DAB (Nacalai Tesque, Kyoto, Japan).

### Immunofluorescence

Immunofluorescence studies were performed by incubation with primary antibodies at room temperature overnight. Secondary antibodies were then added and incubated at room temperature for 2 h. Fluorescence images were obtained using an AxioImager with an AxioCam MRm digital camera (Carl Zeiss, Tokyo, Japan). AxioVision (Carl Zeiss) acquisition software was used to obtain images. For some images, brightness levels were subsequently adjusted using Photoshop (Adobe Systems Japan, Tokyo, Japan). No other image processing was performed. For quantification, the number of immune-positive cells was counted in the somatosensory and motor cortex area (1 mm^2^) of a single coronal section per mouse. The data from four to six animals were statistically analyzed.

### Microarray Analysis

RNA was isolated from the cortices of *Hexb*^−/−^ and *Hexb*^+/−^ mice (n = 4 each) using Sepasol-RNA I Super G (Nacalai Tesque, Kyoto, Japan), and purified using a High Pure RNA Isolation Kit (Roche, Basel, Switzerland). The purity and concentration of total RNAs were determined using an Agilent 2100 Bioanalyzer (Agilent Technologies, Santa Clara, CA, USA). A total of 300 ng total RNA was processed for cRNA amplification, conversion, and labeling according to the manufacturer’s instructions using an Ambion WT expression Kit (Life Technologies, Carlsbad, CA, USA) and an Affymetrix GeneChip WT Terminal Labeling Kit (Life Technologies). Labeled cRNA was hybridized at 45 °C for 17 h with an Affymetrix Mouse Gene 1.0ST Array (Life Technologies). The mouse Gene 1.0 ST Array interrogates a total number of 28,853 mouse genes with 770,317 distinct probes. The arrays were washed in GeneChip Fluidic Station 450, and scanned using a GeneChip Scanner 3000 7 G (Life Technologies). Data collection was performed using GeneChip Operating Software (Life Technologies). Raw data were expressed as CEL files and were normalized using the robust multiarray average method with Expression Console software (Life Technologies). RMA-normalized data was analyzed using GeneSpring GX 11.5 software (Agilent). *P*-values of less than 0.05 were considered significant.

### Quantitative RT-PCR

Total RNA was prepared from whole brains of *Hexb*^−/−^ (n = 6), *Hexb*^+/−^ (n = 5), and *Hexb*^−/−^ *FcRγ*^−/−^ mice at 16 weeks (n = 5). Mouse brains were homogenized on ice in a Dounce homogenizer in Sepasol-RNA I Super G (Nacalai Tesque, Kyoto, Japan) and then treated with DNase (TURBO DNA-free™, Ambion/Life Technologies, Austin, TX, USA) to remove genomic DNA contamination. Quantitative reverse transcription and PCR reactions were performed using an ABI Prism 7500 Fast Real-Time PCR system (Applied Biosystems/Life Technologies) using a TaqMan RNA-to-CT 1-Step kit (Applied Biosystems/Life Technologies), according to the manufacturer’s instructions with the 2^(−ΔΔCt)^ method. 18 S ribosomal RNA was used as an internal control. Primer/probe mixes included *Il-1α* (Mm00439620_m1), *Il-1β* (Mm00434228_m1), *TNF-α* (Mm00443260_g1), *Il-6* (Mm00446190_m1), *iNOS* (Mm00440502_m1), and 18 S ribosomal RNA (Mm03928990_g1) from Applied Biosystems/Life Technologies.

### Identification and Measurement of GM2 and Lyso-GM2 ganglioside (Lyso-GM2) in the mouse brain

The cortices of *Hexb*^−/−^ and *Hexb*^+/−^ mice (n = 5 each) were dissected and homogenized in ice-cold homogenization buffer (320 mM sucrose, 1 mM EGTA, and 5 mM HEPES, pH 7.4). Homogenates were centrifuged at 1000 × *g* for 10 min. Supernatants were spun for 90 min at 13,000 × *g*, and resulting pellets were resuspended in homogenization buffer. After deproteinization by methanol, resulting supernatants were transferred into LC vials for analysis. For GM2 ganglioside analysis, a Myghtysil RP-8GP column (6 × 4.6 mm, Imtakt, Kyoto, Japan) was used to separate the sphingolipids, which were then analyzed by tandem mass spectrometry (LCMS-8040, Shimadzu, Kyoto, Japan) using MRM mode. Lyso-GM2 was analyzed similarly to GM2, but with a COSMOSIL HILIC column (10 × 4.6 mm, Nacalai Tesque, Kyoto, Japan). Data were collected in MRM mode using transitions of m/z 1382.7 → m/z 290.1 (GM2) and m/z 1116.6 → m/z 290.1 (Lyso-GM2).

### Drug treatment

Immunosuppressants (FTY720; 1.0 mg/kg, minocycline; 30 mg/kg, or FK506; 2.0 mg/kg) were intraperitoneally administered daily for 7 days from 3 weeks of age to *Hexb*^−/−^ mice^48^,^49^,^50^,^51^. In the case of rotarod test, drug administration water gel (MediGel Sucralose, Clear H_2_O, Portland, ME) was used to orally ingest FTY720^52^. At an estimated total daily intake of 250 mL/kg, the desired concentration of FTY720 in medicated gel was 6 μg/mL to achieve a dose of 1.0 mg/kg/day. The medicated gel was provided in the cage from 3 weeks of age to 15 weeks and refilled daily.

### Rotarod test

Motor co-ordination was measured using rotarod treadmill equipped with automatic fall detector (Muromachi Kikai Co., Tokyo, Japan). Spindle was 30 mm in diameter and flange-to-flange distance was 57 mm. Mice were placed on the rotarod bar for 4 trials with a 30 min interval between trials. Each trails lasted for a maximum of 6 min. The speed of the rod accelerates linearly from 4 to 40 rpm over 5 min, then was maintained for a further 1 min at 40 rpm. The time each mouse remained on the rotarod was measured for each trial.

### Statistical Analysis

For immunohistochemically stained cell and quantitative RT-PCR data, statistical analyses were performed non-parametrically using a Mann–Whitney *U*-test or a Kruskal–Wallis test (non-parametric ANOVA) followed by a Dunn’s post hoc test for multiple comparisons (StatView for Mac). For ganglioside level data, the unpaired Student’s *t*-test was used (StatView for Mac). For rotarod data, repeated-measures ANOVA followed by Tukey’s post hoc *t*-test was used (StatView for Mac).

## Additional Information

**How to cite this article**: Ogawa, Y. *et al*. FcRγ-dependent immune activation initiates astrogliosis during the asymptomatic phase of Sandhoff disease model mice. *Sci. Rep.* **7**, 40518; doi: 10.1038/srep40518 (2017).

**Publisher's note:** Springer Nature remains neutral with regard to jurisdictional claims in published maps and institutional affiliations.

## Supplementary Material

Supplementary Information
