# Supplementary material for: FcRγ-dependent immune activation initiates astrogliosis during the asymptomatic phase of Sandhoff disease model mice
Source: Sci Rep. 2017 Jan 13;7:40518. doi: 10.1038/srep40518 (PMC5234013; doi:10.1038/srep40518)
Supplement: Supplementary Information [file srep40518-s1.pdf]

SUPPLEMENTARY INFORMATION

FcRγ-dependent immune activation initiates astrogliosis during the asymptomatic phase of Sandhoff disease model mice

Yasuhiro Ogawa[1], Takafumi Sano[1], Masahiro Irisa[1], Takashi Kodama[2], Takahiro Saito[1], Eiri Furusawa[1], Katsutoshi Kaizu[1], Yusuke Yanagi[1], Takahiro Tsukimura[2], Tadayasu Togawa[2], Shoji Yamanaka[3], Khoji Itoh[4], Hitoshi Sakuraba[5], and Kazuhiko Oishi[1*]

[1]Department of Pharmacology, Meiji Pharmaceutical University, Tokyo, Japan,
[2]Department of Functional Bioanalysis, Meiji Pharmaceutical University, Tokyo, Japan,
[3]Department of Pathology, Yokohama City University School of Medicine, Yokohama, Japan,
[4]Department of Medicinal Biotechnology, Institute for Medicinal Research, Graduate School of Pharmaceutical Sciences, The University of Tokushima, Tokushima, Japan,
and [5]Department of Clinical Genetics, Meiji Pharmaceutical University, Tokyo, Japan.

Table S1. Top 50 up-regulated genes in cortices of 16-week-old *Hexb-/-* mice (n=4, *P*<0.05).

Gene accession No.	Gene symbol	Gene name	Fold change
NM_017372	*Lyz2*	*Lysozyme 2*	3.59
NM_011337	*Ccl3*	*Chemokine (C-C motif) ligand 3*	3.43
NM_001081957	*Wfdc17*	*WAP four-disulfide core domain 17*	3.07
NM_021274	*Cxcl10*	*Chemokine (C-X-C motif) ligand 10*	2.87
NM_009977	*Cst7*	*Cystatin F*	2.58
NM_009853	*Cd68*	*CD68 antigen*	2.48
NM_010821	*Mpeg1*	*Macrophage expressed gene 1*	2.41
NM_009779	*C3ar1*	*Complement component 3a receptor 1*	2.32
NM_009780	*C4b*	*Complement component 4B*	2.30
NM_010501	*Ifit3*	*Interferon-induced protein with tetratricopeptide repeats 3*	2.00
NM_013489	*Cd84*	*CD84 antigen*	1.98
NM_011662	*Tyrobp*	*TYRO protein tyrosine kinase binding protein*	1.96
NM_030691	*Igsf6*	*Immunoglobulin superfamily, member 6*	1.92
NM_030720	*Gpr84*	*G protein-coupled receptor 84*	1.91
NM_001077189	*Fcgr2b*	*Fc receptor, IgG, low affinity IIb*	1.90
NM_031254	*Trem2*	*Triggering receptor expressed on myeloid cells 2*	1.87
NM_010277	*Gfap*	*Glial fibrillary acidic protein*	1.86
NM_001111058	*Cd33*	*CD33 antigen*	1.85
NM_011150	*Lgals3bp*	*Lectin, galactoside-binding, soluble, 3 binding protein*	1.84
NM_010130	*Emr1*	*EGF-like module containing, mucin-like, hormone receptor-like sequence 1*	1.82
NM_008534	*Ly9*	*Lymphocyte antigen 9*	1.76
NM_010554	*Il1a*	*Interleukin 1 alpha*	1.69
NM_001166409	*Rbm3*	*RNA binding motif protein 3*	1.68
NM_019549	*Plek*	*Pleckstrin*	1.67
NM_001267695	*Ctss*	*Cathepsin S*	1.65
NM_010185	*Fcer1g*	*Fc receptor, IgE, high affinity I, gamma polypeptide*	1.65
NM_023065	*Ifi30*	*Interferon gamma inducible protein 30*	1.64
NM_139142	*Slc6a20a*	*Solute carrier family 6 member 20A*	1.64
NM_008220	*Hbb-bt*	*Hemoglobin, beta adult t chain*	1.64
NM_007806	*Cyba*	*Cytochrome b-245, alpha polypeptide*	1.63
NM_007572	*C1qa*	*Complement component 1, q subcomponent, alpha polypeptide*	1.63
NM_020008	*Clec7a*	*C-type lectin domain family 7, member a*	1.63
NM_010188	*Fcgr3*	*Fc receptor, IgG, low affinity III*	1.62
NM_008331	*Ifit1*	*Interferon-induced protein with tetratricopeptide repeats 1*	1.60
NM_007574	*C1qc*	*Complement component 1, q subcomponent, C chain*	1.60
AK006938	*1700072H12Rik*	*1700072H12 product:hypothetical protein*	1.61
NM_001083955	*Hba-a2*	*Hemoglobin alpha, adult chain 2*	1.60
NM_013590	*Lyz1*	*Lysozyme 1*	1.60
NM_134158	*AF251705*	*cDNA sequence AF251705*	1.58
NM_021334	*Itgax*	*Integrin alpha X*	1.58
NM_008218	*Hba-a1*	*Hemoglobin alpha, adult chain 1*	1.57
NM_007649	*Cd48*	*CD48 antigen*	1.56
NM_001042489	*Hvcn1*	*Hydrogen voltage-gated channel 1*	1.55
NM_027836	*Ms4a7*	*Membrane-spanning 4-domains, subfamily A, member 7*	1.55
NM_008479	*Lag3*	*Lymphocyte-activation gene 3*	1.54
NM_009777	*C1qb*	*Complement component 1, q subcomponent, beta polypeptide*	1.53
NM_011332	*Ccl17*	*Chemokine (C-C motif) ligand 17*	1.52
NM_025378	*Ifitm3*	*Interferon induced transmembrane protein 3*	1.52
NM_001204910	*AI607873*	*Expressed sequence AI607873*	1.52
NM_013706	*Cd52*	*CD52 antigen*	1.52

Table S2. Top 50 down-regulated genes in cortices of 16-week-old *Hexb-/-* mice (n=4, *P*<0.05).

Gene accession No.	Gene symbol	Gene name	Fold change
NR_004414	*Rnu2-10*	*U2 small nuclear RNA 10*	1.73
NR_029412	*Snora16a*	*Small nucleolar RNA, H/ACA box 16A*	1.71
NM_023842	*Dsp*	*Desmoplakin*	1.68
NM_177068	*Olfml2b*	*Olfactomedin-like 2B*	1.68
NM_138304	*Calml4*	*Calmodulin-like 4*	1.67
NR_002905	*Snora74a*	*Small nucleolar RNA, H/ACA box 74A*	1.66
NM_026956	*Cd209f*	*CD209f antigen*	1.65
XM_003086154	*Vwa3b*	*Von Willebrand factor A domain containing 3B*	1.64
NR_045188	*St18*	*Suppression of tumorigenicity 18*	1.63
NM_029972	*Ermn*	*Ermin, ERM-like protein*	1.58
NR_004439	*Rprl2*	*Ribonuclease P RNA-like 2*	1.54
NM_028390	*Anln*	*Anillin, actin binding protein*	1.54
NM_146257	*Slc29a4*	*Solute carrier family 29, member 4*	1.53
NM_001081078	*Lct*	*Lactase*	1.52
NM_009026	*Rasd1*	*RAS, dexamethasone-induced 1*	1.51
NR_033336	*Snora23*	*Small nucleolar RNA, H/ACA box 23*	1.51
NM_008937	*Prox1*	*Prospero homeobox 1*	1.51
NM_011674	*Ugt8a*	*UDP galactosyltransferase 8A*	1.49
NM_001252552	*Folr1*	*Folate receptor 1*	1.47
NR_026942	*E330013P04Rik*	*RIKEN cDNA E330013P04 gene*	1.46
NM_133238	*Cd209a*	*CD209a antigen*	1.44
NR_028078	*Snora21*	*Small nucleolar RNA, H/ACA box 21*	1.44
NR_046306	*DQ267102*	*SnoRNA DQ267102*	1.44
NM_001100182	*Cyp2j12*	*Cytochrome P450, family 2, subfamily j, polypeptide 12*	1.42
NM_153156	*Stoml3*	*Stomatin (Epb7.2)-like 3*	1.42
NM_178685	*Pcdh20*	*Protocadherin 20*	1.42
NM_015743	*Nr4a3*	*Nuclear receptor subfamily 4, group A, member 3*	1.41
NM_178086	*Fa2h*	*Fatty acid 2-hydroxylase*	1.41
NM_010762	*Mal*	*Myelin and lymphocyte protein, T cell differentiation protein*	1.41
NM_080726	*Rem2*	*Rad and gem related GTP binding protein 2*	1.41
NM_024413	*Plekhf1*	*Pleckstrin homology domain containing, family F (with FYVE domain) member 1*	1.40
NR_046144	*n-R5s136*	*Nuclear encoded rRNA 5S 136*	1.39
NM_011177	*Klk6*	*Kallikrein related-peptidase 6*	1.39
NR_004434	*Rprl1*	*Ribonuclease P RNA-like 1*	1.39
NM_008037	*Fosl2*	*Fos-like antigen 2*	1.39
NR_004412	*Rnu1b1*	*U1b1 small nuclear RNA*	1.38
NM_153520	*Opalin*	*Oligodendrocytic myelin paranodal and inner loop protein*	1.38
NM_010153	*Erbb3*	*V-erb-b2 erythroblastic leukemia viral oncogene homolog 3*	1.37
NM_177624	*Sntn*	*Sentan, cilia apical structure protein*	1.37
NM_026731	*Ppp1r14a*	*Protein phosphatase 1, regulatory (inhibitor) subunit 14A*	1.37
NM_001033349	*Gm410*	*Predicted gene 410*	1.35
NM_001013374	*Lman2l*	*Lectin, mannose-binding 2-like*	1.35
NR_001460	*Rmrp*	*RNA component of mitochondrial RNAase P*	1.35
NM_010338	*Gpr37*	*G protein-coupled receptor 37*	1.34
NM_009398	*Tnfaip6*	*Tumor necrosis factor alpha induced protein 6*	1.34
NM_029001	*Elovl7*	*ELOVL family member 7, elongation of long chain fatty acids*	1.34
NM_025821	*Carhsp1*	*Calcium regulated heat stable protein 1*	1.34
NM_001042715	*Ccdc135*	*Coiled-coil domain containing 135*	1.34
NM_198102	*Tra2a*	*Transformer 2 alpha homolog*	1.33
NM_001161767	*Galnt6*	*UDP-N-acetyl-alpha-D-galactosamine:polypeptide N-acetylgalactosaminyltransferase 6*	1.33

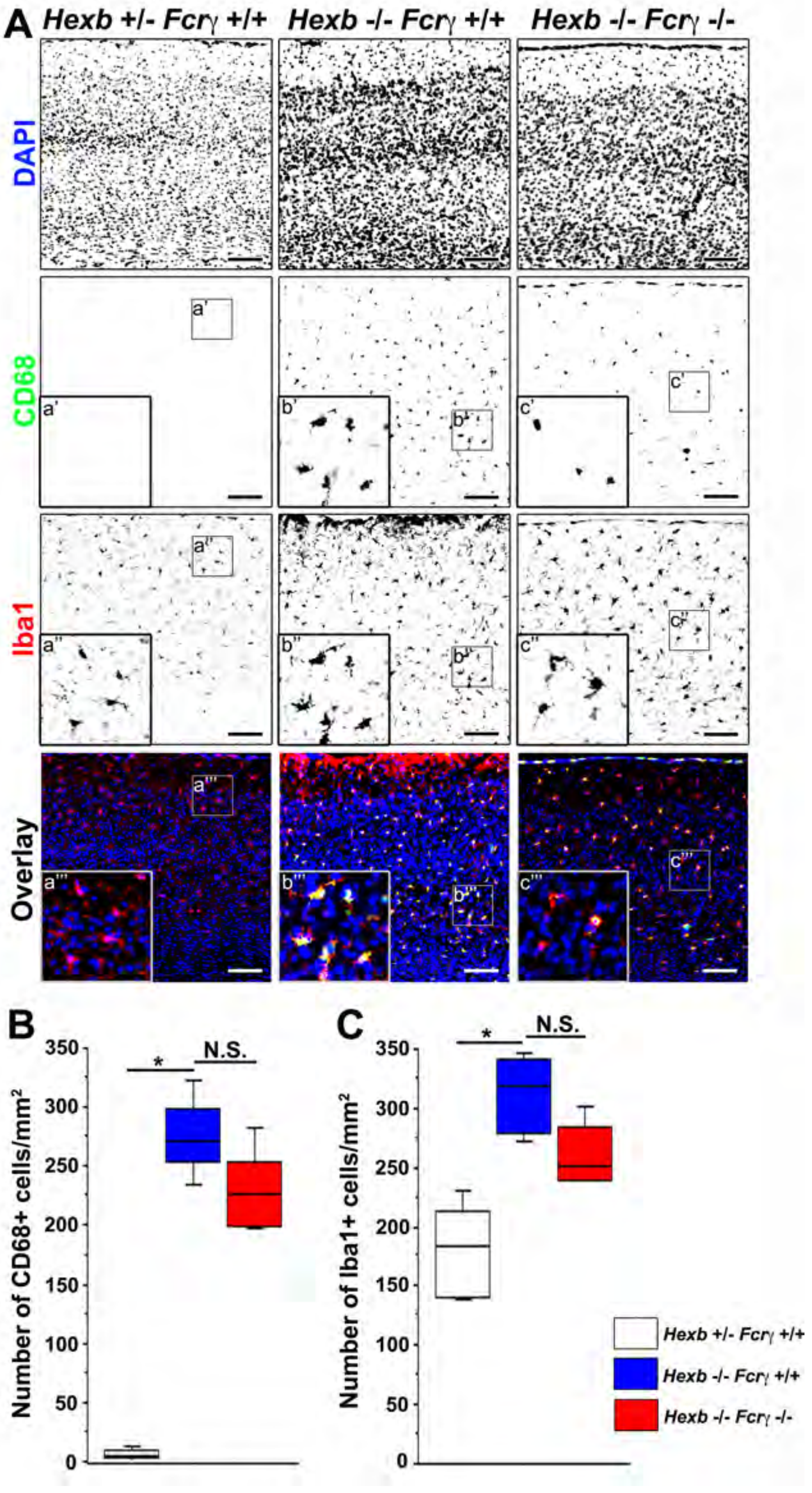

Figure S1. Reduction in microglial activity in cortices of *Hexb*$^{-/-}$ *FcRγ*$^{-/-}$ mice at 16 weeks. *A*, Immunostaining of coronal sections for CD68 (green) and Iba1 (red) in the cerebral cortices of *Hexb*$^{+/-}$ *FcRγ*$^{+/+}$, *Hexb*$^{-/-}$ *FcRγ*$^{+/+}$, and *Hexb*$^{-/-}$ *FcRγ*$^{-/-}$ mice at 16 weeks. Blue represents DAPI staining. Insets (a–c) show magnified views of the boxed regions. Scale bar, 100 μm. *B and C*, Quantitative analysis for the number of CD68+ (B) and Iba1+ (C) cell immune signals in the cerebral cortices of *Hexb*$^{+/-}$ *FcRγ*$^{+/+}$, *Hexb*$^{-/-}$ *FcRγ*$^{+/+}$, and *Hexb*$^{-/-}$ *FcRγ*$^{-/-}$ mice at 16 weeks. Boxes, 25th–75th percentile with the median indicated; bars, 10th and 90th percentiles. Analyzed using a Kruskal–Wallis test (nonparametric ANOVA) followed by a Dunn's post hoc test (n=5). N.S.: difference not significant ($P>0.05$), $*P<0.05$.

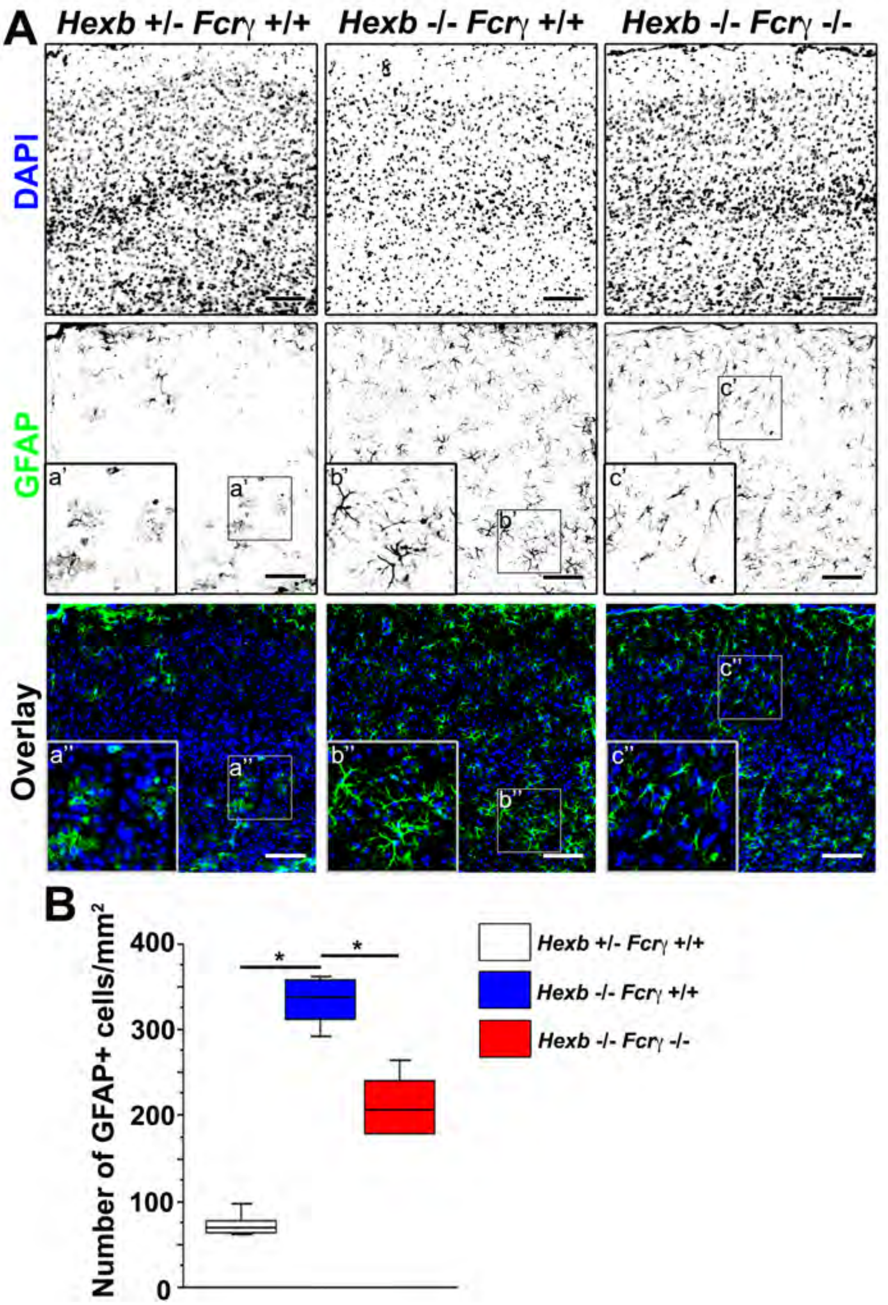

Figure S2. Reduction in reactive astrogliosis in cortices of *Hexb*$^{-/-}$ *FcRγ*$^{-/-}$ mice at 16 weeks. *A*, Immunostaining of coronal sections for GFAP (green) in the cerebral cortices of *Hexb*$^{+/-}$ *FcRγ*$^{+/+}$, *Hexb*$^{-/-}$ *FcRγ*$^{+/+}$, and *Hexb*$^{-/-}$ *FcRγ*$^{-/-}$ mice at 16 weeks. Blue represents DAPI staining. Insets (a–c) show magnified views of the boxed regions. Scale bar, 100 µm. *B*, Quantitative analysis for the number of GFAP+ cell immune signals in the cerebral cortices of *Hexb*$^{+/-}$ *FcRγ*$^{+/+}$, *Hexb*$^{-/-}$ *FcRγ*$^{+/+}$, and *Hexb*$^{-/-}$ *FcRγ*$^{-/-}$ mice at 16 weeks. Boxes, 25th–75th percentile with the median indicated; bars, 10th and 90th percentiles. Analyzed using a Kruskal–Wallis test (nonparametric ANOVA) followed by a Dunn's post hoc test (n=5). **P*<0.05.

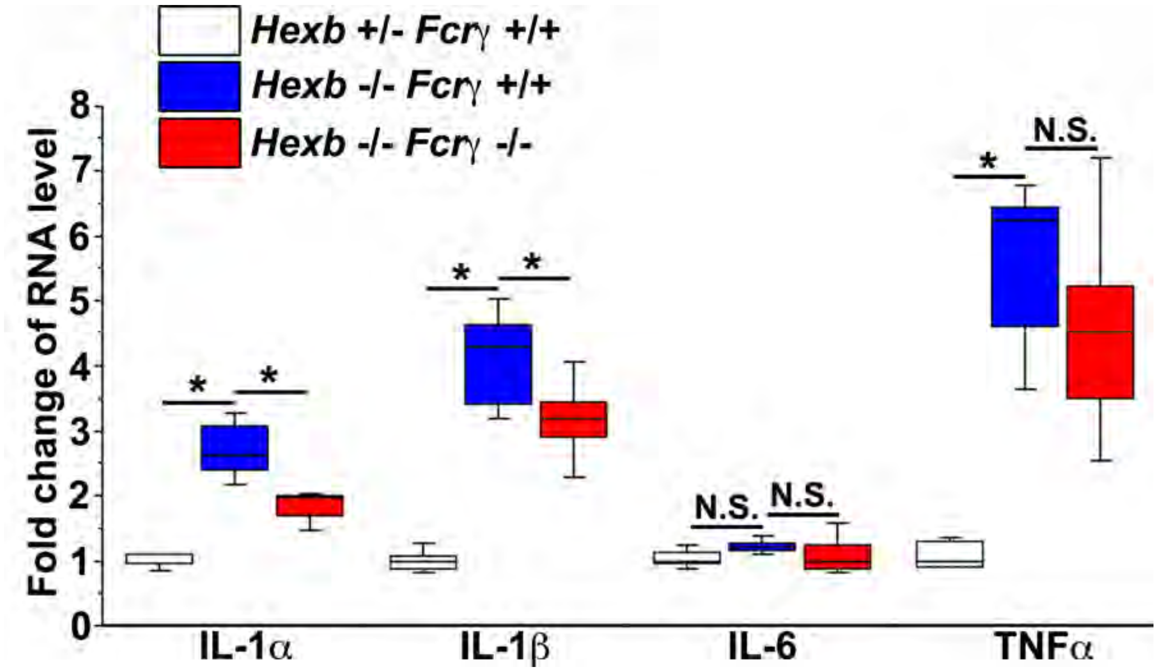

Figure S3. mRNA expression levels were measured by real-time PCR. Expression levels were standardized by those of 18S ribosomal RNA. The values show relative gene expression levels in the cerebral cortices of *Hexb*$^{+/-}$ *FcRγ*$^{+/+}$ (open box), *Hexb*$^{-/-}$ *FcRγ*$^{+/+}$ (blue box), and *Hexb*$^{-/-}$ *FcRγ*$^{-/-}$ (red box) mice at 16 weeks. Boxes, 25th–75th percentile with the median indicated; bars, 10th and 90th percentiles. Analyzed using a Kruskal–Wallis test (nonparametric ANOVA) followed by a Dunn's post hoc test (n=5). N.S.: difference not significant (P>0.05), *P<0.05.

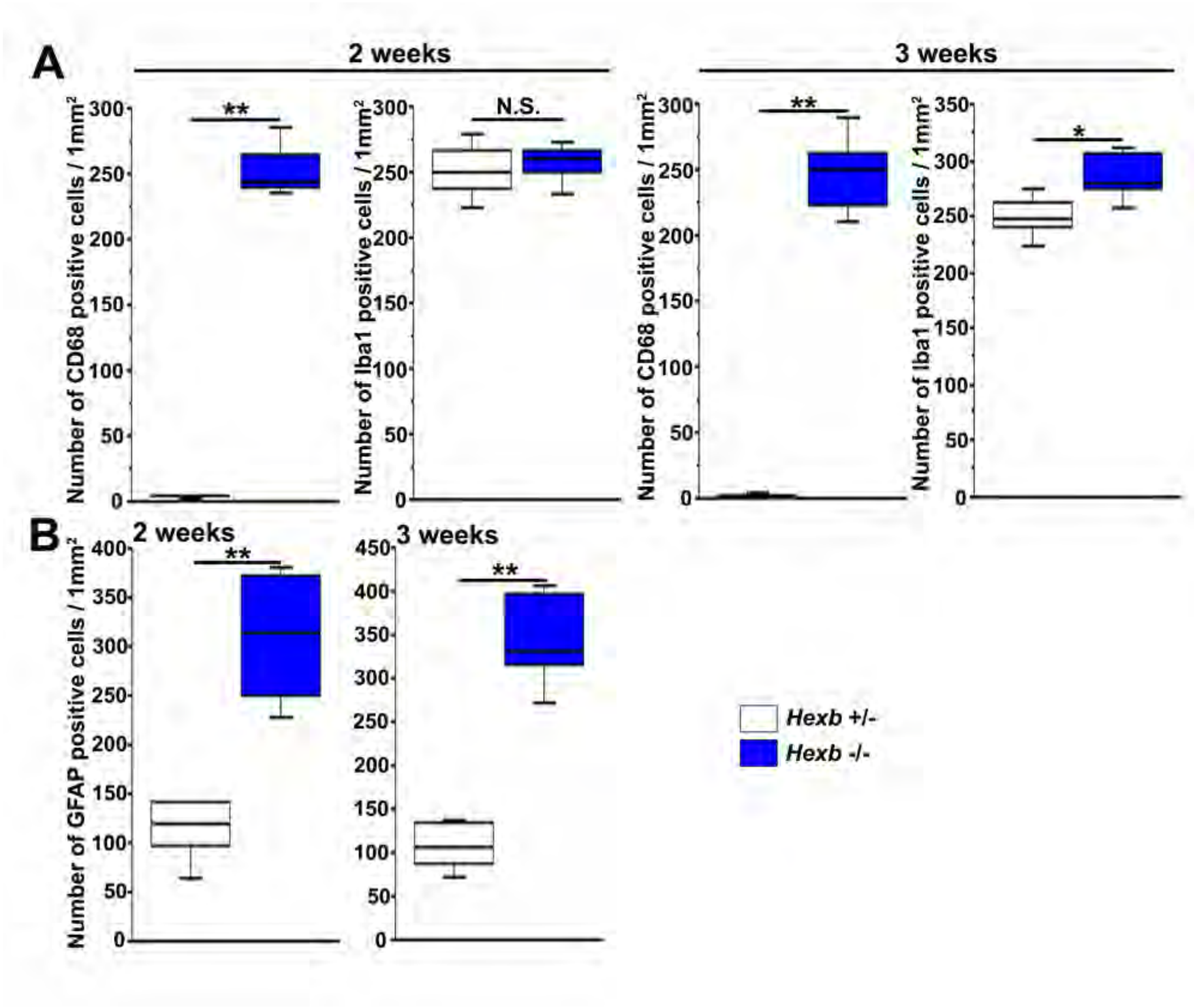

Figure S4. Quantitative analysis for microglial activation and astrogliosis in the cerebral cortices of *Hexb*$^{+/-}$ and *Hexb*$^{-/-}$ mice during development from 2 weeks to 3 weeks. *A*, Quantitative analysis for the number of CD68+ and Iba1+ cell immune signals. Median values of CD68-positive cells per 1-mm^2 section were 4.0 (3.5−5.0) and 244 (239−272) in 2-week-old *Hexb*$^{+/-}$ and *Hexb*$^{-/-}$mice; 1.0 (0.5−3.0) and 251 (218−271) in 3-week-old *Hexb*$^{+/-}$ and *Hexb*$^{-/-}$mice. Median values of Iba1-positive cells per 1-mm^2 section were 250 (233−271) and 260 (244−269) in 2-week-old *Hexb*$^{+/-}$ and *Hexb*$^{-/-}$mice; 247 (235−266) and 280 (269−307) in 3-week-old *Hexb*$^{+/-}$ and *Hexb*$^{-/-}$mice. *B*, Quantitative analysis for the number of GFAP+ cell immune signals. Median values of GFAP-positive cells per 1-mm^2 section were 120 (87−141) and 314 (243−376) in 2-week-old *Hexb*$^{+/-}$ and *Hexb*$^{-/-}$mice; 106 (84−136) and 332 (301−400) in 3-week-old *Hexb*$^{+/-}$ and *Hexb*$^{-/-}$mice. Boxes, 25th–75th percentile with the median indicated; bars, 10th and 90th percentiles. Analyzed using the Mann-Whitney *U*-test (n=5). **P*<0.05, ***P*<0.01. N.S.: difference not significant (*P*>0.05).

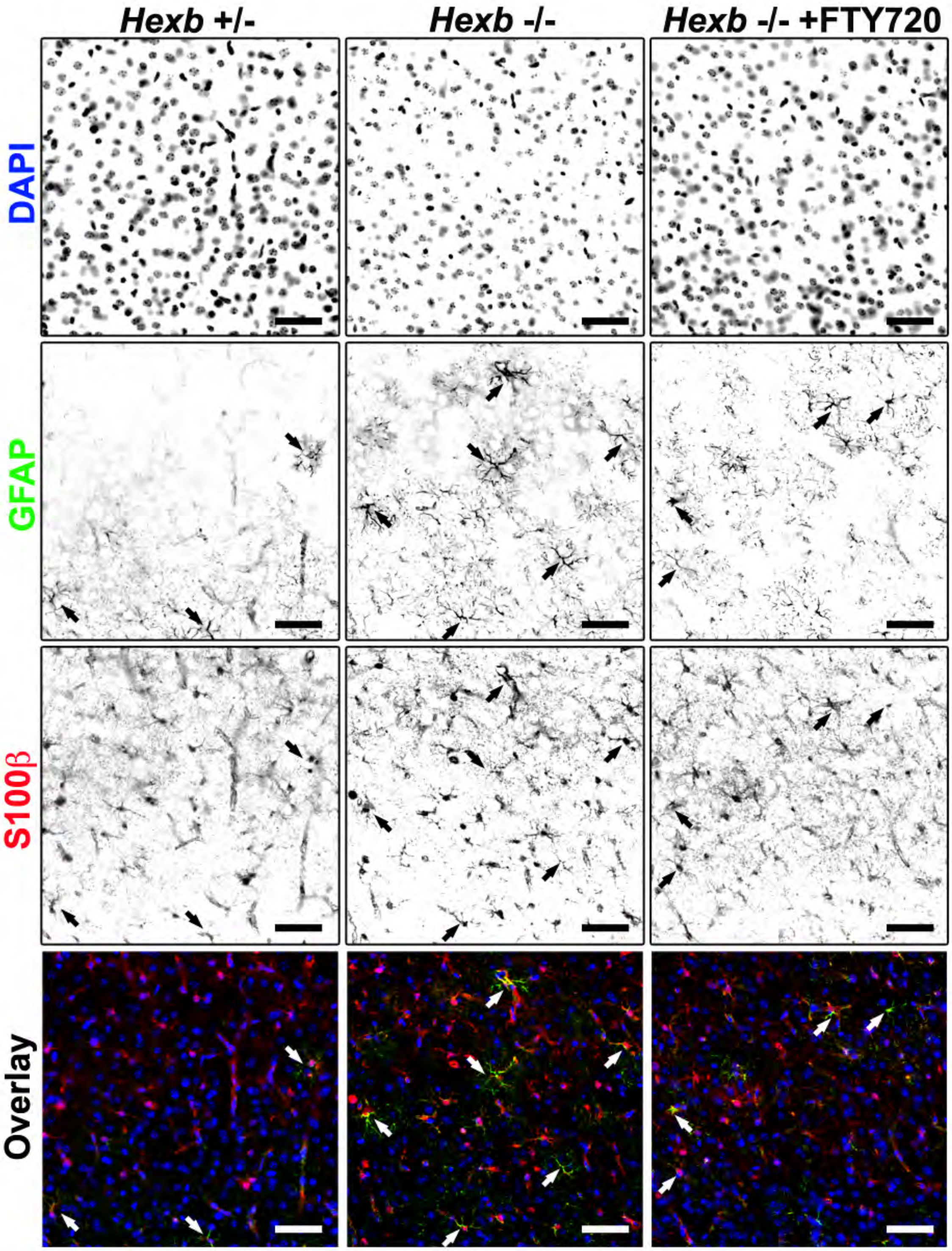

Figure S5. Astroglial avtivation in brains of 4-week-old *Hexb*$^{-/-}$ mice. Immunostaining of coronal sections for GFAP (green) and S100β (red) in the cerebral cortices of *Hexb*$^{+/-}$, *Hexb*$^{-/-}$ and FTY720-treated *Hexb*$^{-/-}$ mice at 4 weeks. Arrows indicate GFAP / S100β double-positive cells. Blue represents DAPI staining. Scale bar, 50 μm.
